# Consequences of COVID-19 pandemics on the mental well-being of general population of Pakistan

**DOI:** 10.1186/s43045-022-00211-2

**Published:** 2022-06-10

**Authors:** Um-e-Kalsoom, Nadia Bibi

**Affiliations:** 1 Department of Psychology, Shaheed Benazir Bhutto Women University, Peshawar, Pakistan; 2 Department of Microbiology, Shaheed Benazir Bhutto Women University, Peshawar, Pakistan

**Keywords:** Anxiety, Depression, COVID-19, Impact of life events, Center of Epidemiologic Studies-Depression

## Abstract

**Background:**

Due to the COVID-19 outbreak, the epicenter is facing transcending psychiatric problems.

To assess the consequences of the COVID-19 pandemic on the mental well-being of the community of Peshawar, Khyber Pakhtunkhwa (Pakistan), a cross-sectional study design was used to find out depression and anxiety after the first wave of the pandemic. A total of 320 willing individuals participated in the study. Convenience sampling technique was used to collect the data. Demographic information along with a semi-structured interview, Hospital Anxiety and Depression Scale, Impact of Life Event Scale-Revised, and Center of Epidemiologic Studies-Depression Scale were used as measures. The participants of this study were bifurcated into affected (*n* = 151) and none affected (*n* = 169) on the basis of the impact of life event cut-off scores.

**Results:**

Data analysis was carried out using *t*-test and simple linear regression analysis. Results of *t*-test showed that the pandemic-affected individuals (47%) reported significantly high on the depression and anxiety scores. The verdicts from simple linear regression analysis further demonstrate a history of psychiatric illness, duration of quarantine, and impact of event predicting depression (*R*^2^ = .15, *p* < .001). For anxiety history of psychiatric illness, the impact of life events were significant predictors (*R*^2^ = .28, *p* < .001) whereas the duration of quarantine, death due to COVID-19, and Impact of Event Scale predicted the center of epidemiological studies for depression (*R*^2^ .48, *p* < .001). The finding of the research study concluded that 47% participated individuals were affected due to the pandemic COVID-19.

**Conclusions:**

The outcome of the study further exhibits that history of previous psychiatric illness, impact of life events, death due to COVID-19, and duration of quarantine are significant predictors of depression and anxiety.

## Background

A novel coronavirus formally known as SARS-CoV-2 flared up in China and spread everywhere throughout the world. Young [1] reported that between January and March 2020, the illness spread to over more than 110 countries and the quantity of cases expanded up to 13-fold. Therefore, World Health Organization (WHO) declared the novel COVID-19 outbreak a pandemic on 11 March 2020 [2, 3]. As per the Johns Hopkins Coronavirus Resource Center [4], there were over 3.6 million affirmed cases and more than 251,898 passing around the world as early as May 5, 2020. To break the procession of disease and control, the pandemic countries started a chain of measures, including nearby and global travel boycotts; restrictions on huge get-togethers; suspension of public vehicles; shutting down of schools, colleges, and business; social separation; stay-at-home requests; and curfews [5].

These restrictions and the uncertain trend of the disease can significantly affect mental well-being and significant impairment of people’s lives. Severe pandemic lockdowns and quarantine are a source of distress since individuals are confined, socially isolated, and facing a high risk of losing their income, as their activities are restricted [6]. According to the authors, factors such as the long duration of quarantine, fears of infection, inadequate information, stigma, or financial loss were related to a higher negative psychological impact. These major stressors can be expected to lead to an increased risk of psychopathology such as anxiety or depression [7, 8]. Current investigations have shown that the COVID-19 pandemic influenced psychological well-being. A review citing investigation of 28 articles highlighted the issue of psychological well-being and exposed the wide-spread occurrence of anxiety, depression, and self-reported pressure related to upset sleep patterns because of the pandemic [9]. Another review called attention to that maladaptive behavior, emotional distress, and defensive reactions as mental responses that can be capable during a pandemic [10]. More specifically, data reported the prevalence of significant post-traumatic stress symptoms and anxiety. Ubiquitously, the entirety of the investigations that have analyzed the psychological issues during the COVID-19 pandemic has revealed that affected people exhibited a number of indications of mental shocks, like emotional agony, sadness, stress, mood swings, temperament, sleep deprivation, hyperactivity problem, post-trauma pressure, and outrage [6, 11, 12].

As far as we could possibly know, no past study investigation in this area (Peshawar) of Pakistan has been conducted to find out not only the prevalence but also the causal factor behind depression and anxiety in the general population during COVID-19. The objectives of this study were to investigate the prevalence of depression and anxiety during the COVID-19 pandemic. An additional objective was to identify causes of depression and anxiety during the pandemic related to the COVID-19 outbreak.

## Methods

World Health Organization (WHO) sample size calculator was used. We assumed a proportion of 30% of the population to have depression and anxiety, at 95% confidence level, and 5% absolute precision, and the sample size calculated was 323 participants. The sample for the present study comprised of both males and females (*N* = 320) ranging in age from 18 to 65 years. All individuals who were meeting the age limit criteria and living in Peshawar city were included in the study. Respondents below the age of 18 and elderly more than 65 years of age; having a serious psychiatric illness (e.g., schizophrenia, mood disorder, and psychosis); or suffering from any chronic physical diseases like cancer, cardiovascular, or chronic kidney disease were excluded from the study. The bedridden adults were also excluded from the study. The consent form described the aims of the study, refusal at any stage to participate in the study, as well as assurance that the said information will be confidential and will be used only for research purposes. The demographic sheet was prepared in order to obtain the following information i.e age, sex, marital status, living system, area, income, education, employment status, To assess the impact of the COVID-19 outbreak the participants were asked the following questions: (a) any history of psychiatric illness, (b) duration of quarantine, (c) know someone to have COVID-19, and (d) death due to COVID-19. To assess post-traumatic stress disorder, the Impact of Life Event Scale-revised (IES-R) comprised of 22 items was developed by Daniel Weiss and Charles Marmar [13]. The IES-R provided a useful tool for assessing the effect of severe stressful events. The higher score indicates the severe impact of an event. The Cronbach alpha reliability in the present sample was .93. Hospital Anxiety and Depression Scale (HADS) was developed to assess depression and anxiety symptoms [14]. The scale comprised of 14 items based on two subscales, i.e., depression and anxiety. The odd 7 items assess anxiety symptoms whereas the even 7 numbers indicate depressive symptoms. The score indicates three categories of depression and anxiety, i.e., mild (8–10), moderate (11–14), and severe (15–21), whereas a score below 7 suggests no depression and anxiety. Center of epidemiological studies- depression (CES-D) was designed to screen current depressive symptoms [15]. The four-point Likert questionnaires comprised of 22 items with potential scores ranging from 0 to 63. A higher score suggests the severity of the depression during the last week. The alpha reliability of the scale in the present study was .91. The sample of the present study comprised of 320 individuals from the general population of Peshawar city. The data was collected during the smart lockdown period (1 September–15 December 2020) from the open market places where both genders were easily accessible. Those meeting the inclusion and exclusion criteria were invited to participate in the study. Convenient sampling technique was used to collect the data. Six volunteer psychologists take part in data collection. These data collectors were given a further 2 days of training for overall assessment protocols, guidelines, the process of informed consent, and questionnaire administration. Ethical approval was sought from the concerned department (Decision no 2211/Dy/ reg/PGMI). Those who were willing to participate in the study were administered a set of questionnaire comprised of demographic information, HADS, IES-R, and CES-D. The participants were briefed about the study objectives and assured about the confidentiality of the information. They further ensured that this information will be used only for the research purpose.

### Statistical analysis

Statistical analysis was performed using SPSS for Windows, V.22.0. Demographic variables such as gender, education, marital status, family status, employment status, and area were reported as frequencies and percentages. Independent sample *t*-test was used to find out affected (depression and anxiety) and none affected (no depression and anxiety). Multiple regression analysis was used to find out the causal factor of depression and anxiety during the pandemic.

## Results

In the current study, data was bifurcated into affected (*n* = 151) and not affected (*n* = 169) group on the basis of impact of life events cut off scores. In the current sample various categories included, none depressed (*n* = 96), borderline depressed (*n* = 115), depressed (*n* = 109), none anxious (*n* = 87), borderline anxiety (*n* = 126), and anxiety sufferers (*n* = 107). Further *t*-test and regression analysis were used to find out the differences and predictors. Independent sample *t*-test displayed considerable differences between the affected and not affected group on CES-D (*t* = − 10.52, *p* < .001), anxiety (*t* = − 7.03, *p* < .001), and depression (*t* = 3.95, *p* < .001). Multiple regression analysis was computed for predicting factors behind developing depression and anxiety. The results showed history of psychiatric illness (*B* = 2.325, *p* < .001, *B* = .325, *p* < .001) and impact of life event (*B* = .048, *p* <. 001) as predictors of depression. Furthermore, a history of psychiatric illness (*B* = 2.2, *p* <.002) and impact of life event (*B* = .087, *P* < .001) were predictors for anxiety, whereas any family member death due to COVID-19 (*B* = 2.18, *P* < .001), impact of event (*B* = .501, *p* <.001), and duration of quarantine (*B* = 1.08, *P* <.025) caused CES-D high scores (Tables 1, 2 and 3).

**Table 1:** Socio demographics of the sample (*N* = 320)

Variables	*N* (%)
**Gender**	
Male	72 (21.8)
Female	248 (74.9)
**Education**	
Illiterate	6 (1.8)
Primary	8 (2.4)
Secondary	58 (17.5)
Above	233 (70.4)
**Marital status**	
Married	74 (22.4)
Single	244 (73.7)
**Family system**	
Joint	151 (45.6)
Nuclear	168 (50.8)
**Employment Status**	
Employed	76 (23.0)
Unemployed	244 (73.7)
**Area**	
Urban	194 (58.6)
Rural	123 (37.)

**Table 2:** Predictors of anxiety among respondents after the first wave of COVID-19

Variable	*B*	95% CI		Sig
		LL	UL	
Constant	4.869	4.043	5.696	0.001
History of psychiatric illness	2.2	0.822	3.57	0.002
Know someone to have COVID	0.348	− 0.451	1.146	0.392
Duration of quarantine	0.121	− 0.159	0.402	0.395
Death due to COVID-19	0.266	− 0.015	0.546	0.063
Impact of event	0.087	0.068	0.106	0.001
*R*^2^ = .281				

Multiple linear regression analysis showing that history of psychiatric illness and impact of life events explained 53% variance for anxiety [*F* (5,314) = 24.540, *p* < .001]

**Table 3:** Predictors of depression using Center of Epidemiologic Study-Depression among respondents after the first wave of COVID-19

Variable	*B*	95% CI		Sig
		LL	UL	
Constant	5.94	3.134	8.745	0.001
History of psychiatric illness	− 0.714	− 5.393	3.965	0.764
Know someone to have COVID	− 1.421	− 4.132	1.29	303
Duration of quarantine	1.088	0.137	2.04	0.025
Death due to COVID-19	2.18	1.228	3.132	0.001
Impact of event	0.501	0.435	0.566	0.001
*R*^2^ = .482				

Multiple linear regression analysis showing that history of psychiatric illness, duration of quarantine, death due to COVID-19, and impact of life events explained 69% variance for depression [*F* (5,314) = 58.544, *p* < .001]

## Discussion

COVID-19 is not only a medical emergency but it smashes all aspects of human life. To reduce the spread of contagious viruses, numerous measures have been taken including quarantine/self-isolation and lockdown. Keeping in view under developed status of the country with limited health facilities Pakistani government announced strict lock down from March till June 2020. The lock-down in Pakistan worked successfully to control the spread of diseases but on the other hand severely impacted the psychological well-being. This study revealed that 47% population was affected from the COVID-19 outbreak. Results of this cross-sectional study are consistent with the prior researches and concluded the adverse psychological consequences in the studied sample of both quarantine and disease outbreaks [16, 17]. The results presented in Table 4 suggest that depression and anxiety were high in the affected groups of the present sample. A comparison on the basis of two cross-sectional studies revealed elevated depression and anxiety levels in adolescents of China after the COVID-19 outbreak [18]. To analyze the impact of COVID-19 on the well-being of health workers, a study conducted in Pakistan also concluded high depression, anxiety, and stress in healthcare workers [19]. Sun et al. [16] also reported alarming psychiatric symptoms (i.e., 46.55% depression, 34.73% anxiety, and 19.56% with suicidal ideation) among university students in China. The reason behind elevated levels of depression and anxiety might be due to the fact that the virus is new and health professionals have no idea how to combat the disease. Coping with the current pandemic is a highly challenging task, and consequently, health professionals strictly warn common people to be away from other individuals, and as a result, a stringent lockdown was forced in almost all over the world. This quarantine/isolation imposed an undesirable impact not only on social, economical, and physical but also on the mental health of individuals belonging to all walks of life.

**Table 4:** Comparison between the affected and non-affected groups on the Center of Epidemiological Studies Depression Scale and Hospital Anxiety and Depression Scale

Variables	Non-affected (*N* = 169)	Affected (*N* = 151)	95% CI			*P*	Cohen’s *d*
	*M* (SD)	*M* (SD)	*t*	LL	UL		
CESD	17.242 (10.091)	32.72 (15.35)	− 10.52	− 18.37	− 12.58	0.001	1.19
H. anxiety	7.03 (3.34)	9.78 (3.64)	− 7.03	− 3.51	− 1.97	0.001	0.78
H. depression	6.36 (3.10)	7.77 (3.26)	− 3.95	− 2.11	− 0.71	0.001	0.44
df = 318							

The result of the *t*-test shows that there is a significant difference between the affected and non-affected groups on CES-D and Hospital Anxiety and Depression Scale

The current study investigated a previous history of psychiatric illness, duration of quarantine, impact of life events, and death due to COVID-19 as significant predictors of depression and anxiety. Results depicted in Table 5 suggest that history of psychiatric illness, duration of quarantine, and the impact of life events significantly cause depression. Anxiety is caused by a previous history of psychiatric illness and the Impact of an Event Scale. Whereas factors significantly responsible for CES-D are the duration of quarantine (*P* <.025), any family member’s death due to COVID-19 (*p* < .001), and the impact of the event (*p* <.001). A previous history of psychiatric history as a strong causal factor of depression and anxiety during pandemic period might be possible due to the fact that such individuals may have experienced limited medical assistance. Therefore, not only did quarantine and the pandemic period have their effect, but also previous psychiatric symptoms flourished due to poor medical adherence. Qiu et al. [20] also reported that 35% population shows psychological symptoms due to pandemic outbreaks. Ozdin and Ozdin [21] also reported that women’s gender and a previous history of psychiatric and physical illness living in an urban area are significant predictors of depression and anxiety. Another study corroborates a multifarious connection between fear, stress, and anxiety to develop depression during the pandemic in undergraduate students.

**Table 5:** Predictors of depression among respondents after the first wave of COVID-19

Variable	*B*	95% CI		Sig
		LL	UL	
Constant	4.922	4.143	5.702	0.001
History of psychiatric illness	2.325	− 1.187	0.319	0.001
Know someone to have COVID	− 0.434	0.083	0.611	0.257
Duration of quarantine	0.347	0.029	0.065	0.010
Death due to COVID-19	0.11	− 0.154	0.375	0.412
Impact of event	0.048	1.025	3.625	0.001
*R*2 = .153				

Multiple linear regression analysis showing that history of psychiatric illness and impact of life events explained 39% variance for depression [*F* (5,314) = 11.369, *p* < .001]

## Conclusions

The findings of our study showed that 47% population get affected from COVID-19 and reported depression and anxiety. Further results suggested the predictors of depression and anxiety are a history of psychiatric illness and impact of life events, whereas predictors of depression according to CES-D are the duration of quarantine, any family member’s death due to COVID-19, and impact of life events.

## Data Availability

Upon request will be shared.

## Abbreviations

IES-R: Impact of Events Scales-Revised
HADS: Hospital Anxiety and Depression Scale
CES-D: Center of Epidemiologic Studies-Depression Scale
